# The ethical adoption of artificial intelligence in radiology

**DOI:** 10.1259/bjro.20190020

**Published:** 2019-11-29

**Authors:** Keshav Shree Mudgal, Neelanjan Das

**Affiliations:** 1 King’s College Hospital Foundation Trust, London, UK; 2 East Kent Hospitals Foundation Trust, Canterbury, UK

## Abstract

Artificial intelligence (AI) is rapidly transforming healthcare—with radiology at the pioneering forefront. To be trustfully adopted, AI needs to be lawful, ethical and robust. This article covers the different aspects of a safe and sustainable deployment of AI in radiology during: *training, integration and regulation*.

For training, data must be appropriately valued, and deals with AI companies must be centralized. Companies must clearly define anonymization and consent, and patients must be well-informed about their data usage. Data fed into algorithms must be made AI-ready by refining, purification, digitization and centralization. Finally, data must represent various demographics.

AI needs to be safely integrated with *radiologists-in-the-loop:* guiding forming concepts of AI solutions and supervising training and feedback. To be well-regulated, AI systems must be approved by a health authority and agreements must be made upon liability for errors, roles of supervised and unsupervised AI and fair workforce distribution (between AI and radiologists), with a renewal of policy at regular intervals. Any errors made must have a root-cause analysis, with outcomes fedback to companies to close the loop*—*thus enabling a dynamic best prediction system.

In the distant future, AI may act autonomously with little human supervision. Ethical training and integration can ensure a "transparent" technology that will allow insight: helping us reflect on our current understanding of imaging interpretation and fill knowledge gaps, eventually moulding radiological practice. This article proposes recommendations for ethical practise that can guide a nationalized framework to build a *sustainable and transparent* system.

## Introduction

Artificial intelligence (AI) has been aiding healthcare since its first use for dosing antibiotics in the 1980s.^1^ With the current explosion of machine learning, development by companies such as Google DeepMind^2^ and BenevolentAI,^3,4^ AI is set to rapidly transform healthcare. Within radiology, most development has focused on abnormality detection^5^ and future development is envisioned in molecular imaging, radiogenomics and whole population cancer screening.^6^

Few algorithms however, have been clinically tested or implemented: DeepMind Optical Coherent Tomography (OCT) analysis at Moorfields Eye Hospital^7^ and within radiology, Mammogram Intelligence Assessment (MIA) at United Lincolnshire Hospitals NHS Trust^8^ are two sparse examples. All deployed AI cases have made individual agreements and as such, there is no national regulation framework or guidance on the ethics of such adoptions.

To be trustfully adopted, AI needs to be lawful, ethical and robust.^9^ AI integration must ensure maximum benefit for patients and users, whilst actively ensuring non-maleficence. This can be brought about by fair and non-biased development of collaborative AI between healthcare and industry—an intelligence that can be supervised to be safely and effectively integrated into clinical application.

Innovators in AI are unfamiliar with healthcare policies around medical ethics and research regulation and it is the healthcare industry’s responsibility to educate and ensure an ethical adoption keeping in mind AI’s clinical, practical and emotional impact.^10^

This article covers key ethical issues involved in AI training, integration and regulation and aims to guide recommendations for a national framework for AI with ethical values.

## What is ARTIFICIAL INTELLIGENCE APPLIED TO RADIOLOGY?

The fathers of AI, Minsky and McCarthy, described AI as any task carried out by a machine which, if done by humans, would require intelligence.^1^

AI is developed using *machine learning (ML*): where large sets of data (such as chest X-rays) are fed into an algorithm with some knowledge about the data (such as pneumonia or not) which the algorithm processes and trains on. The algorithm then applies its training to make a prediction, *i.e.* pneumonia or not on "test" images. As the algorithm optimizes its parameters to improve diagnostic accuracy, it *learns* to be a better prediction model.^11^

**Figure 1.**
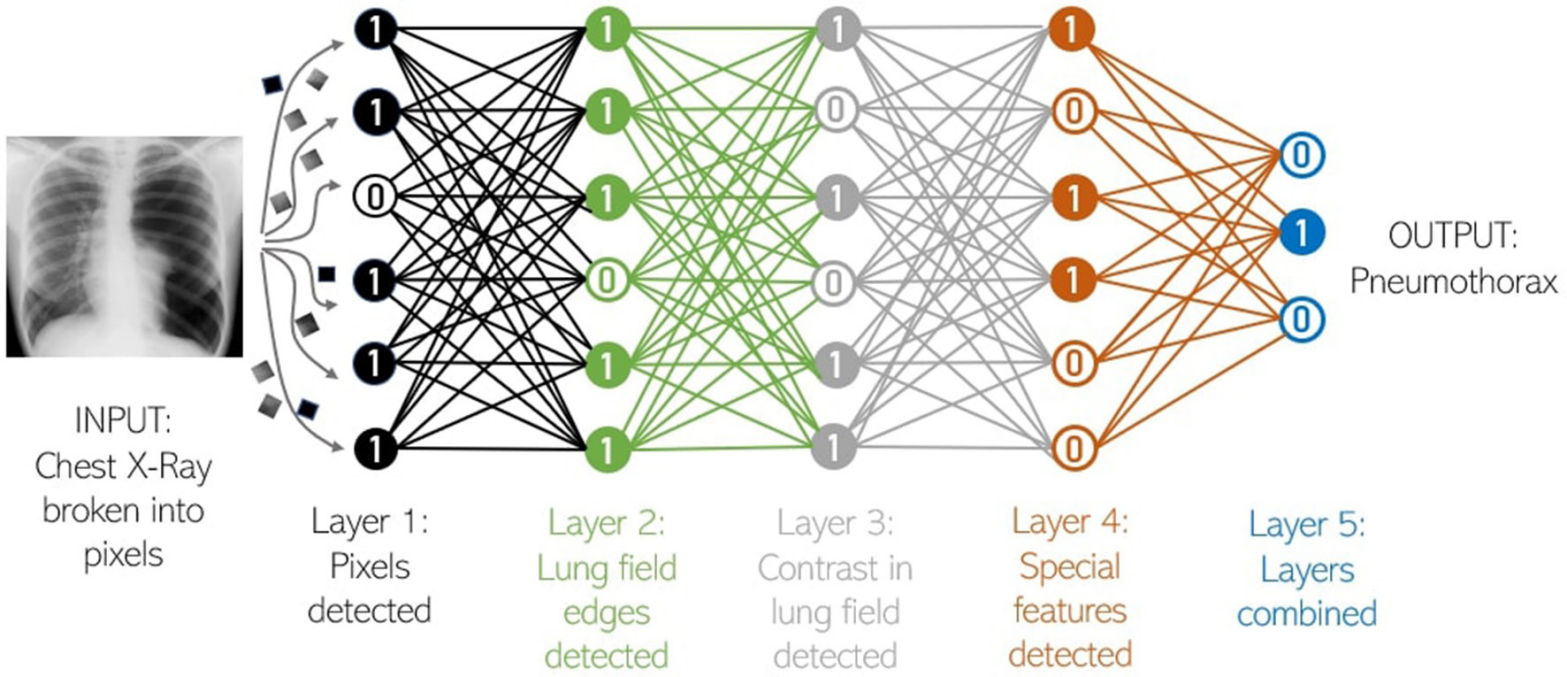
Deep convolutional neural networks use simple processing "neurons" that connect in layers, with signals from neurons merging to a convoluted kernel at the next layer. Each layer weighs information from kernels and computes image features that are believed to be of importance in making the prediction or diagnosis of interest. Signals are transmitted between layers and the algorithm identifies the best combination of these image features for classifying the image and produces its output.^1,2^ Furthermore, a process of “back propagation” makes minute alterations in individual neurons so the network learns to produce the correct output. Once the system has learnt from multiple images, it becomes an expert at recognizing a likely outcome such as a "pneumothorax." *Adapted from: ‘New Theory cracks open the black box of deep neural networks’. Wired (10 August 2017):* https://www.wired.com/story/new-theory-deep-learning/ *[accessed 15/10/2018]*

ML uses *neural networks*, and more recently (specifically for visual imagery) *convolutional neural networks:* these are inspired by the visual cortex of the human brain and assume that inputs have a geometric relationship (similar to rows and columns in images) (see Figure 1).

Earlier algorithms involved only 3–4 layers but recently >200 layers have been developed to bind together and form complex problem-solving systems known as *deep learning (DL*). This has allowed algorithms to make predictions without requiring specified labelled features. DL finds features by itself—adapting along the way and this makes it a very accurate and fast predictive system.^11^

### Why is radiology pioneering AI in healthcare?

**Figure 2.**
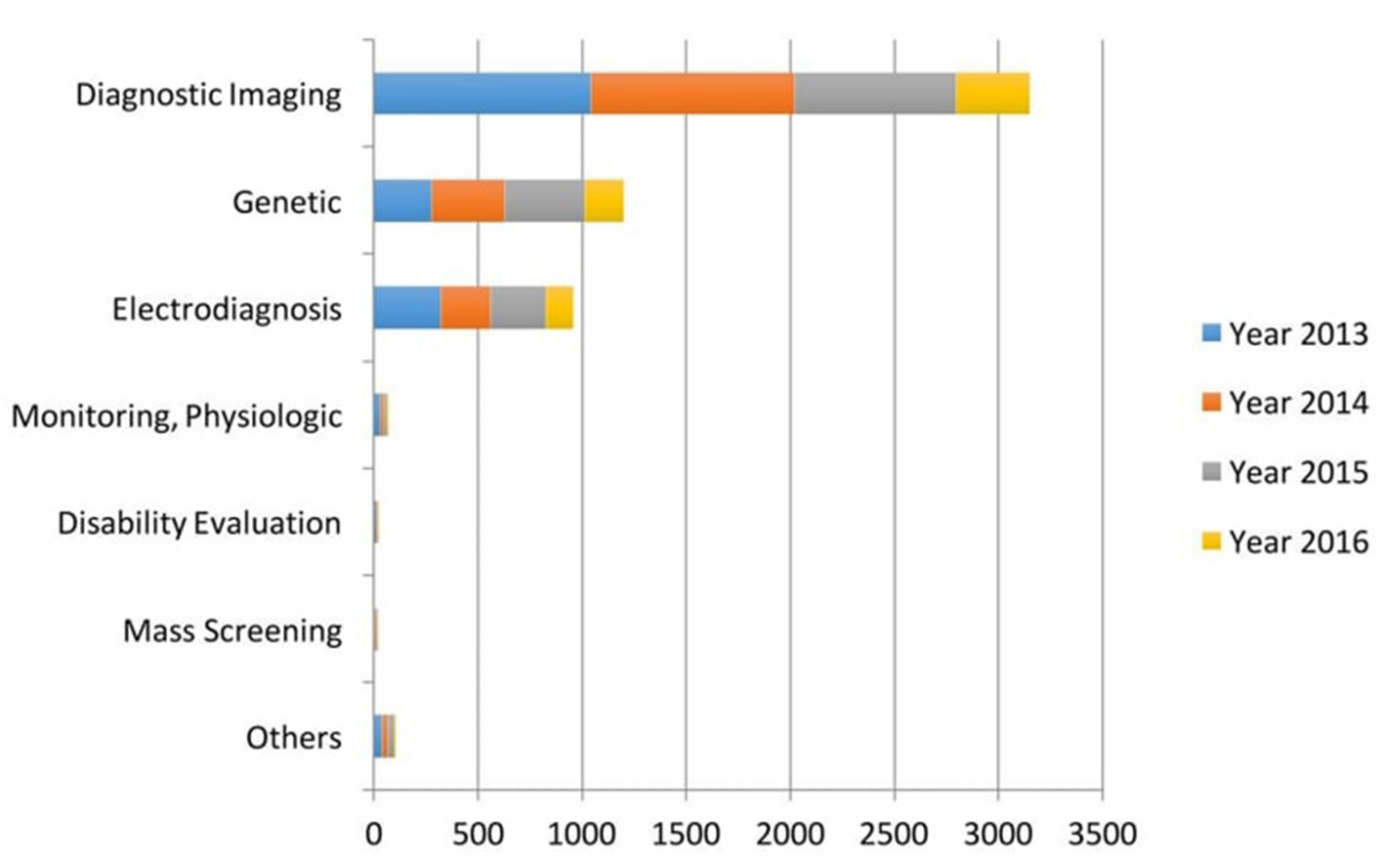
A comparison of diagnostic techniques used in recent in AI studies. *Acquired from "Artificial intelligence in healthcare past present and future by Jiang et al." Permission for re-use has been granted*

Radiology has always been the most digitally informed specialty and was the first to adopt computer science.^12^ Medical imaging has been the most researched field in ML (See Figure 2),^13^ and over the last 10 years, publications in AI radiology have gone up from 100*–*150 to 700–800 per year^12^ MRI and CT-scans account for more than 50% of this research with neuroradiology being the leading specialty followed by musculoskeletal, breast, cardiovascular, urogenital, lung and abdomen.^12^

AI development has relied on large data sets^14^ being available via storage solutions of PACS (Picture Archiving and Communication System) and metadata DICOM (Digital Imaging and Communications in Medicine)—systems that have archived millions of digitized images over the last 30 years^15–17^ and have embedded information that has propelled DL. Furthermore, open access to large annotated datasets such as ImageNet has bred healthy AI competition and growth.^14^

Recent success in AI image classification has also propelled radiological DL with improved algorithm accuracy from 77% in 2011 to 97% in 2016; and current accuracy at level with or exceeding human performance.^14^

### What current AI is being applied in radiology?

Current radiological AI adopted by the NHS is sparse. Dartford and Gravesham NHS Trust have deployed the Behold.ai red dot prioritization platform to prioritize abnormal chest X-rays, aiding workflow^18^ and MIA has been approved for trials at two trusts in the Midlands: acting as a second-reader for breast cancer screening.^19^ Similarly, East Kent Hospitals University NHS Foundation Trust are soon piloting a chest X-ray prioritisation and audit system using qure.ai.

Overseas, in the United States, *Viz.ai* has been deployed to rapidly analyze CT angiographies for detecting strokes and producing cerebral perfusion maps—these are urgently sent to on-call stroke teams for a streamlined stroke workflow and synchronized care.^20^ In China, *Infervision* is being used for lung cancer screening and detecting haemorrhagic strokes by over 300 hospitals. Furthermore, their *InferScholar* Centre is being used for carrying out medical research.^21^

Whilst not many AI systems have been approved for use, there are plenty in the pipeline. DeepMind are working with University College London Hospital and Imperial College London to create tools for improving efficiency of head and neck cancer radiotherapy, and detecting breast cancer, respectively. Hospital patient-flow is being addressed by a recent AI partnership between the Turing Institute and University College London Hospital.^22^ Nvidia is working in King’s College London to work towards neuroimaging and cancer solutions^23^ and Royal Surrey County Hospital and DeepMind AI are working together using their "OPTIMAM" mammography database to improve the quality of reporting of screening mammograms.^24^

Overall, the majority of AI being developed aims to assist radiology in image detection and segmentation, precision medicine linking genomic and imaging data, substituting in double-read screening programmes and rapid detection in emergency situations.^6^ Furthermore, in the next few years, radiology is deemed to be most benefitted from workflow efficacy algorithms aiding: reporting prioritization, real-time image quality assessment, creating study protocols for follow-ups and increasing image quality from noisy low-quality images (in effect performing faster MRIs and reducing CT scan radiation doses).^14^

It is clear that, with the current pace of AI development, adoption on a large scale is inevitable and for this adoption to be sustainable, it requires robust oversight and guidance that will ensure an ethical acceptance by its users and patients.

## Ethics of adoption

### Training and development

#### The value of patient data

As remarked by Lord Henley in the House of Lords, the NHS has “quantity and quality data that other countries don’t have."^1^ Companies such as Roche have paid up to $1.9 billion for healthcare data on 2 million cancer patients^25^ and extrapolated to the NHS (where 1.4 million patients are seen daily^26^) its data are worth trillions of pounds, if not more.

So how much are patient data really worth? The value of data is defined not by its face value but its intended usage. Hypothetically, companies could enter an open marketplace and the highest bidder could acquire the most patient data. In fact, past AI deals have been made under unregulated agreements where individual NHS trusts have made inconsistent financial deals. Not only does this harbour a fluctuating marketplace, it also puts patient-data at risk.^1^

This begs several ethical questions:

§ what is the highest monetary value for data?

§ Can “enough money” ever justify unethical use of data?

§ Is it even fair to “sell” patient data as a concept?

Viewed as a model of symbiosis, data-sharing can allow the NHS to “freely-trade” with technology from AI companies: such as the (non-AI) partnership between DeepMind and Royal Free London Foundation Trust. The trust received 5 years free usage of their Steams app in exchange for patient data for app development.^1^ A “transactional” model is another agreement*—*exemplified by Oxford NHS Trust and Drayson where the trust took a £5 million equity stake in Drayson, thereby sharing in any potential profits, as well as, risks.^27^

If and when trusts do undergo financial agreements with AI, the Department of Health and Social Care (DoHSC) ask AI companies to speculate prospective effectiveness and overall economic impact of their product. They also ask companies to consider data value post-initial training and any potential cost savings to help arrive at an overall value of the AI algorithm^10^ that can be used to formulate a balanced financial agreement.

Indeed, the UK government via Innovate UK, is propelling the concept of *symbiotic partnership* with a large sum of funding being made available for access to shared healthcare data. The ISCF or Industry Strategy Challenge Fund is Innovate UK’s strategy to allow leverage of existing healthcare data to fuel early predictive diagnostic tools. Over £210 million is made available to allow the NHS, academia and industry to collaborate and deliver precision medicine. This includes funding for AI/ML in radiological technology such as MRI and ultrasound scan.^28^

### Centralized data

A solution to eliminate the inconsistencies in AI deals would be centralization of shareable data with a single platform of contact and expertise. Indeed, the health secretary, Matt Hancock recently launched NHSX as part of his "Tech Vision" in July 2019, which brings together the DoHSC, NHS England and NHS Improvement to drive the digital transformation of healthcare. This £1 billion a year investment is projected to allow a single point of accountability where policies for data sharing and transparency can be developed to centrally orchestrate deals and distribution of NHS data sets.^29^

Within radiology, the Royal College of Radiologists (RCR) has proposed an "NHS AI Institute"^30^ and Dr Harvey, BRAIN (British Radiology Artificial Intelligence Network)^31^ with similar objectives. With the commencement of ISCF, many national AI hubs centralizing data have emerged. These include the London Medical Imaging and Artificial Intelligence Centre*—*a consortium of major London university hospitals building AI transformation to improve patient pathways for dementia, heart failure and cancer.^32^

Scotland has its own consortium of fifteen partners building the International Centre for Artificial Intelligence Research in Digital Diagnostics (iCAIRD)*—*focusing on stroke, chest X-ray readings and breast cancer screening.^33^

Oxford University is utilizing its cloud-based Oxford Big Data Institute to “pipeline innovation” and focus on cancer, heart disease and metabolic health through its National Consortium of Intelligent Medical Imaging.^34^

A centralized place of access to information such as NHSX, in conjunction with emerging AI hubs as a result of large government-based funding is the way forward. Proposals should be set up and contribute to populate a large single interoperable repository of health imaging, enabling the development, testing and validation of AI-based health imaging solutions to improve diagnosis, disease prediction and follow-up of the most common forms of cancer and chronic diseases.

Venturing overseas, the European Commission Horizon 2020 are funding a “Global BioImaging Project” that is working towards a sustainable imaging infrastructure that would allow global access to medical imaging solutions. This project aims to build a common virtual platform for training and analysis of imaging technologies that would allow easy access to cutting-edge research to all the global scientific community.^35^

### AI-ready data

Before imaging data can be fed into ML, it needs to be made “AI ready.” In its current format, data are largely inaccessible with a lot of noise and omissions. This required refining by rigorous and time-consuming processes^31^ to make it useful. The DoHSC ask for AI companies to have built-in data quality evaluation to aid this and ensure noisy data don’t harm development.^10^

Furthermore, imaging data need purification. Before the report attached to an image is used as gold-standard for training, the image should be double or triple read by radiologists. This would minimize error rates and built-in biases, so AI can train on data sets that would produce outcomes most representative of its population.

Lastly, for data to be fully centralized, they require digitization. Although most radiological imaging exists in this format, the associated reports and patient documentation does not*—*NHSX hopes to catapult this digitization over the next few years.^29^ These conditions, along with an easily accessible storage solution such as cloud platforms (such as used by cardiac imaging AI by Arterys^36^ and limited chest X-ray and head CTs interpretation by qure.ai)^37,38^ will make imaging data *ready* for ML.

### Patient engagement with data

Before data are made accessible for AI manipulation, they need to be approved for usage. It should not be assumed that patients have good understanding of AI and thus, truly *informed* to provide consent. A recent Royal Society survey showed that only 9% of the respondents knew what the term "machine learning" meant.^1^ Patients need to be empowered with information on their data usage under the Data Protection Act 2018^39^ ; with guidance on how AI works, what problems it will solve (with clearly defined outcomes), when it is used and if (or when) a human is involved in the decision-making process.

Alternatively, patient data are used regularly in hospital audits and quality improvement projects without explicit consent. It is implied that audits benefit clinical practice and consequently, the patient. In the case of radiological imaging, once data are anonymized, how imperative is it to gain explicit consent?*—*AI algorithms will, prospectively, benefit the patient’s health.

To address this, EU General Data Protection Regulation ask for all data used in processing to be opt-in. Recital 32 specifically sets a high bar for opt-in consent, and states that silence, inactivity or pre-ticked boxes do not constitute consent.^40^

Paradoxically however, the DoHSC suggest all companies processing healthcare-data to comply with the national *opt-out* policy by 2020^10^ (as is currently being administered by NHS Digital).^41^ Furthermore, they guide that if anonymized with the Information Commissioner’s Office (ICO) code of practice^42^ and confidential, data are exempt from consent in most cases. To see whether opt-out is applicable, DoHSC suggest data controllers to utilize data flow maps.^10^

Regardless of whether companies use an opt-in or opt-out scheme, they must adhere to Caldicott principles by giving legitimate justification for accessing data and using minimum data required.^43^ Companies must also specify the level of anonymization and importantly, specify the data regulations they are implementing.^10^ Furthermore, if companies share unanonymiszd data with third parties, a formal explicit consenting process with a "right to be forgotten (data erasure) principle" should be practised.^44^

Overall, the consenting process needs to be promoted in the public realm for patients to understand the consequences of opt-in and opt-out strategies. Special attention should be paid to equity of access-to-information in deprived areas and demographics with English not the first language.^45^ Additionally, if used, consent forms should be easy to understand with "Key Facts" summaries displayed appropriately. The recently established "AI council" could provide a good medium for information dissemination by collaborating with the media and distilling information around AI and data use.^46^

The challenge lies in striking the right regulatory balance between the beneficent consequences of access to patient data and protecting the individual from abuse.

### Anonymization and confidentiality

The General Data Protection Regulation (and its UK implementation, the Data Protection Act 2018^39^), ask for all personal data to be either anonymized or pseudonymized before being processed. Anonymization is where personal data are irreversibly modified and the aim is to prevent identification. Pseudonymization, on the other hand, is where patient identifying information is removed but is stored away securely and can be accessed later for reidentification.^47^

For data-driven AI, the DoHSC guide for data to be preferably anonymized under the ICO code of conduct.^48^ However, this code has not been updated since introduction of the new Data Protection Act in 2018, and would benefit from revising what aspects of patient data are non-negotiable and what may conceal vital information when anonymized. Whilst names, date-of-births and patient ID numbers may not aid much, co-founders such as age, gender, ethnicity and comorbidities may help interpret a person’s imaging. For example, a person’s name will not aid much to diagnosing osteoporosis, but gender, ethnicity and age will. Furthermore, in terms of DL, inputting the patient’s surname may indicate ethnicity to the algorithm and this could aid better diagnostic accuracy to the specific patient-group. AI may benefit from taking revealed genetic and environmental factors into the DL algorithm, but equally has the risk of inherent bias and confidentiality breach.

For a "fully informed" medical prediction system, some believe that outcomes need to be linked back to a patient’s history.^49^ This would allow an unrivalled test of technology, but can only be achieved with pseudonymization or non-anonymization. This type of data usage can be approved under specific regulations if clear benefits of data and results is justified.^10^ It does however, pose the risk of deanonymization with vulnerability to social media and advertising firms. Uniquely to radiology, imaging data from head and face CT scans may also be reconstructed to produce surface rendered images which, if fed into facial recognition software can distinguish individuals.^49^

To safeguard from this, data holders need strong cyber security. This should ideally incorporate audit trails: transparent, immutable and verifiable systems, where patient data accessed by private-companies at any single instance can be traced and data are invulnerable to retrospective manipulation. Blockchain technology could provide an answer to this level of data confidentiality.^50^

On another note, as the younger "digitally aware" generation freely shares (once perceived unshareable) health data across social media, the tolerance of current risks and meaning of privacy will inevitably evolve.^51^ At least in the near future however, companies using data must keep in mind patients’ best interests and comply with the Caldicott principle of "the duty to share information being as important as the duty to protect patient confidentiality."^43^

### Building an inclusive system

AI technology has recently been scrutinized for failing to incorporate diversity into its training. Notably, cases of facial-recognition not differentiating Chinese faces^52^ and African names being characterized as "unpleasant" have been published.^53^ This highlights *inherent bias* which has been described as the single greatest threat of data-driven technology by the DoHSC.^10^ The issue is not that AI is inherently racist or sexist but that many AI algorithms have been developed by a mainly white male population, with data on mainly white male populations.^53^ Clearly, in healthcare such cases must only be outliers and to tackle this there are several initiatives, such as the Ada Lovelace Institute and "Human-Centred AI" at Stanford University ensuring that data used for development are gathered from all demographics.^53,54^

### Benevolent AI

The Asilomar principles state that AI must "benefit and empower" all of humanity.^55^ There are examples of this benevolent practice such as publicly available data sets on bone X-ray^56^ and chest X-rays.^57^ This sharing mindset is essential for breeding competition amongst companies to catapult AI technology so that it can be most beneficial for the clinician on the future.

### Recommendations for the national framework for training AI

1. Collaborations between industry, academia and the NHS must share patient data in a symbiotic manner to bring about an overall benevolent outcome for the patient
2. Government-funded proposals must be set up to create a single large interoperable repository of health imaging enabling the development, testing and validation of AI-based health imaging solutions
3. Any monetary value for patient data must be standardized via a centralized platform with view of investing any profits made, into developing healthcare technology further
4. Imaging data need to be made AI-ready by digitization, cleaning, purifying, labelling and easily accessible storing on cloud-based platforms. AI companies must have built-in data quality evaluation to ensure noisy data are removed.
5. AI companies must give legitimate justification for access to data and specify data regulations they are implementing. If unanonymized data are used to share to third parties, formal explicit consent must be practised with a "data erasure" principle.
6. Companies must use an opt-out consenting service for use of healthcare-data under the DoHSC guidance. If confidential and anonymized under the ICO code, to be exempt from consent.
7. Bodies such as AI Council must promote public awareness of data usage by AI and dispel the consenting process*—*keeping the equity to access of information in mind
8. Data can be anonymized or pseudonymized depending upon use justified by AI companies. Where applicable the latest ICO code of conduct must be followed and justification should be clearly specified by the company
9. Strict national guidance must be developed to define aspects of patient information that can be safely pseudonymized. This requires a tightly controlled audit trail that is invulnerable to retrospective manipulation, something that blockchain technologies may potentially offer
10. Data used in training and development must represent the widest spectrum of demographics with an evergrowing data set to breed the least inherently biased AI
11. Once data are AI-ready, it must be ethically shared to benefit and empower the maximum number of users and patients.

The introduction of AI technology into everyday radiological practice needs thorough planning with frameworks developed to standardize clinical efficacy, governance and medicolegal protection*—*these are covered below.

## Integration

### Built-in safety

Developing AI from its current infancy to an automated system in healthcare is likely to be an iterative process dotted with growth spurts and disruptive events. As narrow AI systems learn and predict outcomes of imaging tests, there is bound to be a large number of false negative and positive results needing correction. Eventually with repetitive supervised learning (akin to a medical student’s teaching), this bracket of false results can be closed and AI, within limited remits, may outperform a radiologist.

However, before achieving this stage, it is important to build a safe system that works in *partnership* with clinicians in an *efficient* way. This lies in the hands of radiologists who need to reinvent themselves as "radiologists-in-the-loop."^6^ From the very conceptualization of an AI system, radiologists can be involved as medical officers guiding institutions and companies in the right direction to target the most relevant and topical clinical decision-making tools. Later, they can act as consultants providing continuous supervision to AI bodies, who can observe incorrect outcomes, flag them up, co-ordinate with ML engineers to investigate root of the issue and feedback into the deep learning process.^6^ In practice, radiologists can regularly audit and supervise AI tools, and investigate medical errors to feedback to AI companies. Such integration would allow a safe transition from current *individual* intelligence to *augmented* radiology intelligence^58^ where a system works autonomously but with human supervision and guidance to enhance its counterpart’s practice.

Some radiological AI products such as MIA by Kheron Medical have been approved under the European CE mark and are being implemented in the NHS.^8^ AI falls under the medical devices bracket and to be safely integrated, the DoHSC suggest products be regulated by a health authority such as Medicines and Healthcare products Regulatory Agency by 2020.^1,59^

This requires companies to be transparent about strengths and limitations of their data and to contextualize data with the algorithm outcome*—*so that differences such as diseases between demographics is accounted for. Companies must also specify whether algorithms in practice are the same as used in development, so safer versions can be integrated. Furthermore, if possible, companies must specify the algorithm’s methodology, disclose whether supervised and demonstrate how outcomes have been validated.^10^ Overall, this allows for a safe system to be integrated, and for safety to be sustainable, radiologists are crucial. They must actively participate as the human authority in the machine learning pipeline*—*initially (as data scientists) for creating validated data sets for training ML models and then (as consultants) for developing new models and testing products.

However, to ensure clinical efficacy and safety is materialized, framework on accountability and liability is required. This will be covered later under *Regulation*.

### Will AI take over radiology?

The grandmaster of deep learning, Geoffrey Hinton remarked that AI may make radiologists redundant.^60^ Recently, the AI system, CheXNeXt claimed to detect a multitude of pathology on chest X-rays at expert radiologist level^61^ and other systems have claimed accuracy superseding radiologists at detecting malignant lung and breast cancers.^62,63^ These collectively beg the question of whether AI will put the radiologist out of a job.

On the other hand of a technological revolution, there has been a 3.2% annual increase in radiological imaging, with the NHS processing 41 million images per year.^64^ This demand is generated by an ageing population, increased multimorbidity and increased screening; and has meant a 30% increase in the radiologist’s workload over the last 5 years.^65^ The RCR has consistently warned of workforce shortages with "red-alerts"^66–69^ and there is an estimated deficit of 1000 consultants in the NHS.^65^ AI can close this workforce gap by carrying out time-consuming, low value, mundane and repetitive tasks and streamline workflow to enable radiologists more face-to-face interaction with patients and increase productivity in: multidisciplinary meetings, interventional procedures, verification of reports, education, policy making and complex clinical decision-making*—*tasks that at least in the near future, can’t be automated. If used as a double-reader, it can also reduce subjectivity (inter- and intraready variability)*—*another recently raised issue^70^ and the adverse effects of reporter fatigue.

Whilst in the short-term, the question may not be of replacement but requirement in radiology, in the long term, AI may replace the radiologist’s role in certain areas. This will only be balanced by growth in other sectors such as in AI medical consultants and medical data scientists.^6^ For this, it is important for AI companies and the NHS to be welcoming to healthcare staff changing career paths and to consider fair workforce distribution. Automation from AI is most set to take over repetitive tasks^71^ and according to the "O-ring principle" of workforce automation, as the radiologist does a smaller proportion of the work, their work is only set be more *valued*.^6^

### Ownership

There is a difference between owning raw data and processed complex data from ML. Processed data are more valuable as it can equate to profit from sharing, reprocessing and further development. It allows the owner to develop and implement AI into practice*—*and more importantly dictate how safety and clinical efficacy is integrated.

Who owns data at different stages*—*is it the NHS, the AI company or the patient? Furthermore, who owns the algorithm once it has used processed data? (initially belonging to the NHS). There are examples that specify ownership for data, but not algorithm, such as NHS trusts in partnership with DeepMind who declare the NHS to hold ownership: DeepMind has a data guardian who is to destroy all data on completion of contract.^72^

Going forward, collaborations must clearly outline ownership of data and algorithms at different stages of use, keeping in mind, the accountability of safe implementation ownership instils.

### Recommendations for the national framework for integration of AI

1. Radiologists to actively participate in the ML development and integration as data scientists and consultants to supervise training and feedback
2. Radiologists to supervise AI companies in ensuring that projects target the most clinically relevant and topical medicine
3. AI systems must be approved under the MHRA by 2020, by exercising transparency regarding: data limitations, outcomes, supervision and validation
4. AI must be welcomed, and radiology must pioneer its use in the healthcare community. Radiologists to actively integrate AI in their departments and learn how to work alongside, to augment practice and reduce workflow crisis
5. As repetitive tasks are automated, industry must be willing to offer roles such as medical data scientists and clinical consultants to healthcare staff
6. NHS and industry to agree upon ownership of AI algorithm and, data at different stages of AI use*—*allowing accountability for a clinically efficient and safe integration

## Regulation

### Who will be liable?

With time as AI gains autonomy, medical application of AI imaging interpretation may no longer be in the radiologist’s hands. The first duty of a doctor is to "do no harm" and to continue this virtue, AI must be validated to be safe, accurate and infallible before being used autonomously.^6^ With automation, there will be need for clarity around legal implications for inappropriate clinical outcomes. For example, who will be liable if AI wrongly reports “no significant abnormalities” but a tumour goes amiss. Will it be the company, the healthcare organization employing the company’s AI or indeed the radiologist?

For the foreseeable future, a *radiologist-in-the-loop^6^* can regularly supervise and audit AI implementation; keeping legal liability and responsibility within remit of the radiologist and employing organization. It can be argued however, that companies profiting from commercial value of AI systems should be jointly and severally liable. In practice this might prove difficult to administer, but a financial pool of AI-related medical errors could be set up where approved AI companies contribute. This would establish pre-defined compensation payments for different levels of proven consequences for medical errors*—*ensuring a swifter resolution for patients and consequently reducing burden on the medicolegal system. As ever, organizations should comply with the duty of candour.^73^

To learn from medical errors, a root-cause analysis system is required*—*with transparent application to outcomes that are incorrect, biased or discriminate against population subsets.^49^ The current format of departmental errors and discrepancy meetings would need adjusting with formation of a potential multidisciplinary AI team involving clinicians, data-scientist and ML engineers.^12^ Any errors determined to be involving AI systems would need to be fed-back to engineers who would in-turn complete the feedback loop by ensuring that appropriate adjustments have been made. These errors could be logged into a national database, much like those held by the MHRA^74^ and this could help determine patterns on a larger scale.

There is however a problem in applying the root-cause analysis to the "black box" of AI: a current knowledge gap in understanding explicitly how an input becomes an AI output.^75^ To combat this, Kohli and Geis suggest "version control" where changes in the development of software are tracked and mistakes can be retrospectively traced back to a specific point in software development.^49^ This could be applied to AI using blockchain technology^50^ and specific steps leading to incorrect outcomes could be isolated and amended to improve quality control.^49^

Furthermore, to demystify the "black box," companies have strategically amended their AI systems. DeepMind have split OCT analysis into two separate consecutive neural networks*—*with observation of consecutive outcomes allowing ophthalmologists to gain insight into AI reasoning at different steps.^7^ Barzilay, is also devising AI where overt reasoning steps are integrated in mammogram analysis for detecting early breast cancer.^76^ Furthermore, an "Explainable Artificial Intelligence" program is underway by the United States military to decipher AI reasoning in defence technology.^76^

As AI increases influence on healthcare decisions in the future, rules around the decision-making process and legal indemnity (including national frameworks for medical defence companies) need to be modified. The RCR asks for a regulatory system that protects both patients and doctors: by defining the professional responsibilities of doctors using AI and management of risk associated with AI tools.^30^

### Trust in AI

Whilst we may believe that we live in an informed society, the media doesn’t always represent the true nature of AI. From films about robot apocalypses^77^ to videos of AI robots on YouTube predicting the end of the human race,^78^ there is an overall fear of AI. Moreover, there are adversarial attacks,^79^ job displacement fears and "death by algorithm" headlines.^80^

Security and privacy hacking is a major fear with personal data being misused.^81^ Within healthcare, DeepMind and NHS Royal Free Hospital were recently under investigation for their "Streams" app. An ICO investigation concluded that patients were misinformed about their data usage with lack of clarity and openness leading to misinformed consent and patients being unable to opt-out.^82^ The Royal Free has since taken measures to rectify this, with one being an opt-out form online.^83^ "Streams" has continued to work for Royal Free as an app that identifies patient at risk of acute kidney injury and is to be piloted at the Imperial Trust after local rigorous governance processes.^84^

This is a positive example of learning from our mistakes and working to reduce the overall fear of AI. If we continue this and keep a transparent relationship between the public, industry and healthcare, we can tip the scale towards a positive impact of AI, at least within healthcare, and pave a safe way for AI augmentation.

### Emotional trust

It is widely agreed that a doctor’s judgement, creativity or empathy can never be replaced, and although AI can suggest diagnoses and treatment prognoses, the "radiologist-in-the-loop" should have the final verdict.^6,30^ This is especially true in cases such as deciding between oncological treatment and commencing palliative care.^59^ At a recent AI conference at the Institute of Engineering and Technology, the prospect of AI possessing *human values* was considered. Human values such as empathy and kindness are dynamic and constantly change*—*what was socially acceptable in the 16th century isn’t anymore and vice versa. It is unlikely that "human values or experience" can be taught to an AI system but, it is more likely that AI builds its *own values*.^85^ This would be a processed output of influences by its teachers and the ongoing socioeconomic and political climate fed into the system. Perhaps advanced AI could *mimic* empathy or in-fact have such profound understanding of treatment effects that presenting them would be as clear as a doctor*—*rendering AI equally as trustworthy? Moreover, most patients don’t read figures or records of a doctors’ performance but follow their intuition*—*and with repetition, this instils trust. If AI can inspire this trust, the doctor’s role may inevitably change.

**Figure 3.**
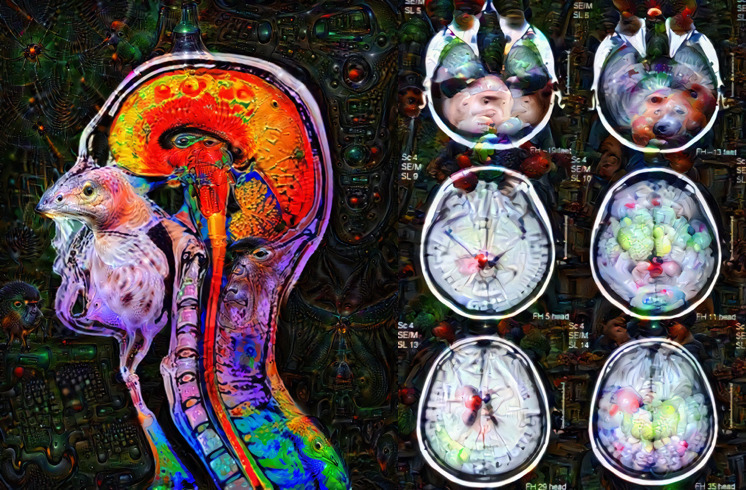
A Deep Dream Al generated images of MRI heads MRI heads were fed into the AI algorithm that produced outputs based on its own black box interpretation of the image. *Developed using “Deep Dream Generator”,* https://deepdreamgenerator.com/ *[accessed 25/10/2018]*

In the future, AI is destined to become more powerful than the human mind, which may in turn, become the limiting factor. Doctors may no longer make diagnoses but will ensure that AI recommended diagnoses are relevant and meaningful to the patient. We will require truly “interpretable AI” that can converse with humans to explain its reasoning.^76^ In fact, compared to its human counterpart, AI may become the more transparent intelligence*—*one that can be audited, interrogated and have knowledge gaps filled.^86^ Pande describes the current "black box" as a feature rather than a drawback*—*allowing reflection on current thinking through observation of outcomes. An example of black box interpretion is expressed by Deep Dream generated images (see Figure 3). This black box may change the way radiologists currently think and decipher patterns in imaging. Furthermore, it may help shed light and redefine what being "human" really means.^86^

### Recommendations for the national framework for regulating AI

1. Industry and NHS must agree upon who has liability for errors made whilst using AI for specific functions. This should be outlined in the agreement from the outset with renewal of policy whenever there is a paradigm shift in AI technology.
2. Role specific to unsupervised AI and AI augmenting the radiologist must be outlined. Responsibility appointed to the radiologist using AI must be fair and justified. This must be renewed on an annual basis and during significant advancements in AI technology
3. In the near future, radiologists to make the final verdict whilst using AI, keeping responsibility in the clinician’s hands
4. For medical errors, a root-cause analysis to be performed using technology that allows specific points in the decision-making process to be delineated. There errors must be logged into a national database to determine any large-scale patterns
5. Industry and NHS to create an AI-related medical errors financial pool, where pre-defined compensation is established for varying levels of medical errors.
6. When errors happen, establishments must always comply with duty of candour
7. We must learn from our mistakes and these should be welcomed to improve the overall quality and delivery of AI, thus building the foundation of a healthy partnership with AI

## Conclusion

There has been a planetary alignment in recent years of massively increased computing power, algorithm training, hardware and software development. This has allowed an exponential growth of AI in almost all aspects of life. We are amid a fourth industrial revolution which reaches far deeper into the fabric of humankind that any before it. Thought leaders both for and against AI are essentially negotiating and creating mechanisms for us to embrace, accept and trust AI.

This article covers key ethical issues with recommendations towards a national framework that if implemented with transparency, accountability, explicability and fairness can ensure healthcare AI to be moulded into a *benevolent* rather than malevolent technology: not only for radiology, but also for other medical specialties.
